# Robotic hernia repair II. English version: Robotic primary ventral and incisional hernia repair (rv‑TAPP and r‑Rives or r‑TARUP). Video report and results of a series of 118 patients

**DOI:** 10.1007/s00104-021-01479-6

**Published:** 2021-08-10

**Authors:** Johannes Baur, Michaela Ramser, Nicola Keller, Filip Muysoms, Jörg Dörfer, Armin Wiegering, Lukas Eisner, Ulrich A. Dietz

**Affiliations:** 1 Department of Visceral, Vascular and Thoracic Surgery, Cantonal Hospital Olten (soH), Baslerstraße 150, 4600 Olten, Switzerland; 2 Department of General, Visceral and Vascular Surgery, Cantonal Hospital Baden, Im Engel 1, 5404 Baden, Switzerland; 3 Department of Surgery, AZ Maria Middelares, Buitenring Sint-Denijs 30, 9000 Ghent, Belgium; 4 Department of General, Visceral, Transplant, Vascular and Pediatric Surgery, University Hospital Wuerzburg, Oberduerrbacher Str. 6, 97080 Wuerzburg, Germany

**Keywords:** Umbilical hernia, Incisional hernia, Primary ventral hernia, Minimally invasive, Retrorectus mesh, Linea alba, Umbilikalhernie, Inzisionalhernie, Primär ventrale Hernie, Minimalinvasiv, Retrorektus Netz, Linea alba

## Abstract

Endoscopic management of umbilical and incisional hernias has adapted to the limitations of conventional laparoscopic instruments over the past 30 years. This includes the development of meshes for intraperitoneal placement (intraperitoneal onlay mesh, IPOM), with antiadhesive coatings; however, adhesions do occur in a significant proportion of these patients. Minimally invasive procedures result in fewer perioperative complications, but with a slightly higher recurrence rate. With the ergonomic resources of robotics, which offers angled instruments, it is now possible to implant meshes in a minimally invasively manner in different abdominal wall layers while achieving morphologic and functional reconstruction of the abdominal wall. This video article presents the treatment of ventral and incisional hernias with mesh implantation into the preperitoneal space (robot-assisted transabdominal preperitoneal ventral hernia repair, r‑ventral TAPP) as well as into the retrorectus space (r-Rives and robotic transabdominal retromuscular umbilical prosthetic repair, r‑TARUP, respectively). The results of a cohort study of 118 consecutive patients are presented and discussed with regard to the added value of the robotic technique in extraperitoneal mesh implantation and in the training of residents.

**Video online:**

The online version of this article (10.1007/s00104-021-01479-6) includes three videos and a fact sheet/OP checklist (Supplement Material 1).

## Abstract (de)

Die endoskopische Versorgung von Umbilikal- und Inzisionalhernien hat sich in den vergangenen 30 Jahren an die Limitationen der konventionellen laparoskopischen Instrumente angepasst. Dazu gehört die Entwicklung von Netzen für die intraperitoneale Lage (intraperitoneales Onlay-Mesh, IPOM) mit antiadhäsiven Beschichtungen; allerdings kommt es bei einem beträchtlichen Teil dieser Patienten doch zu Adhäsionen. Minimal-invasive Verfahren führen zu weniger perioperativen Komplikationen, bei einer etwas höheren Rezidivrate. Mit den ergonomischen Ressourcen der Robotik, die abgewinkelte Instrumente anbietet, besteht erstmals die Möglichkeit, Netze minimal-invasiv in unterschiedliche Bauchdeckenschichten zu implantieren und gleichzeitig eine morphologische und funktionelle Rekonstruktion der Bauchdecke zu erreichen. In diesem Videobeitrag wird die Versorgung von Ventral- und Inzisionalhernien mit Netzimplantation in den präperitonealen Raum (robotische ventrale transabdominelle präperitoneale Patchplastik, rv-TAPP) sowie in den retrorektalen Raum (r-Rives bzw. robotische transabdominelle retromuskuläre umbilikale Patchplastik [r-TARUP]) präsentiert. Es werden die Ergebnisse einer Kohortenstudie an 118 konsekutiven Patienten vorgestellt und im Hinblick auf den Mehrwert der robotischen Technik in der Extraperitonealisierung der Netze und in der Weiterbildung diskutiert.

## Background

Although primary ventral hernias and incisional hernias are distinct entities with correspondingly different indications, perioperative challenges, and noncomparable recurrence prognosis, the two entities are to a certain degree surgically managed in a similar manner. Thus, it is important to analyze both subgroups separately.

There are a wide variety of surgical approaches available:

- The periumbilical approach with preperitoneal mesh insertion (preperitoneal umbilical hernia mesh plasty [PUMP]) [1],
- Open approaches to the retrorectus or to the intraperitoneal onlay mesh (IPOM) position and
- Minimally invasive procedures, either laparoscopic (IPOM, with or without hernia gap closure) or transumbilical as E/MILOS (endoscopic/mini to less open sublay) [2, 3].

Patients with increasing age and higher body mass index (BMI) benefit most from minimally invasive procedures because the risk of complications is lower. However, data show that the recurrence rate is greater with laparoscopic IPOM than with open morphologic and functional reconstruction [2]. In IPOM meshes, in addition to postoperative pain, adhesions to the bowel and, in rare cases, bowel erosion may occur [4]. Paraphrasing the first sentence of Patricia Highsmith’s novel “Ripley’s Game” (1974), it can be postulated that the perfect surgery does not exist because as long as there are mankind and science, there will be idiosyncrasies and new procedures.

Robotics opens new paths: It allows technically easy access to the different layers of the abdominal wall compared to conventional laparoscopy and integrates the advantages of open procedures (fewer recurrences) with those of minimally invasive procedures (fewer complications); thereby the layers can be reconstructed morphologically and functionally, and sufficiently large meshes can be implanted. Robotics has noticeable advantages in the workflow of the operation compared to conventional minimally invasive procedures: Ergonomics, degrees of freedom of the instruments, image stability and immersion view are just some of them. If necessary, lower intraperitoneal pressures can be used in cardiopulmonary stressed patients, as the ports (as in lift laparoscopy) keep the abdominal wall elevated.

In this article, we describe the results of a controlled cohort study with robotic preperitoneal (robotic ventral transabdominal preperitoneal hernia repair [rv-TAPP]) and retrorectus mesh positioning (r-Rives and robotic transabdominal retromuscular umbilical prosthetic hernia repair [r-TARUP], respectively), detailing the surgical steps and demonstrating them in the accompanying videos [5]. This video article is the second of a series of three articles on robotic hernia surgery; part I describes inguinal hernia care [6].

## Indications

The indications for endoscopic robotic repair of primary ventral and incisional hernias are similar in principle to those for conventional laparoscopic procedures and also depend on the patient’s risk profile [2, 4, 7]. In obese patients or with known rectus diastasis, the robotic approach has the advantage over open procedures (the PUMP procedure, for example) that asymptomatic additional findings are also treated [1]. The guidelines recommend mesh implantation for umbilical hernias with a diameter greater than 1 cm [7]. Symptomatic umbilical hernias in obese patients, in patients with high intra-abdominal pressure and epigastric hernias—as well as incisional hernias with a diameter of up to 7 cm—are good indications for the robotic surgery approaches described in this article. According to the etiology, primarily ventral hernias (< 4 cm) are treated with mesh implantation in the preperitoneal space (e.g., rv-TAPP), since there are hardly any scarring adhesions of the peritoneum to the hernia ring. Primary ventral hernias > 4 cm in diameter and incisional hernias (< 7 cm in diameter), on the other hand, are more likely to be treated with mesh implantation in the retrorectus space (e.g., r‑Rives or r‑TARUP). In the case of hernias with a width of more than 8 cm, a robotic transversus abdominis release (r-TAR) seems to be indicated (see part III of this series in *Der Chirurg*, in preparation).

## Patient information

The minimally invasive procedure and the use of the surgical robot are presented. Postoperative complications such as postlaparoscopic shoulder pain, postoperative bleeding, seroma formation, and the occurrence of chronic pain or skin numbness are generally discussed. Positioning on the operating table with the option of spinal extension is addressed. In the case of a slim body, a bulge may form in the area of the skin over the hernia repair, which is very likely to smooth out completely within the first 3–6 months postoperatively.

The puncture site of the Veres needle on the left subcostal and shaving of the abdomen and right thigh (for the neutral electrode) are addressed. The extension of the procedure to the entire linea alba as well as the extension of the procedure from the rv-TAPP to the retrorectus space (r-Rives or r‑TARUP) is left optional depending on the intraoperative findings of the surgeon’s assessment. The available results of conventional reparations are cited as the expected recurrence rate (approximately 2–8% at 5 years). Implantation of a nonabsorbable, flat, large-pored mesh (magnetic resonance imaging [MRI]-visible if appropriate), with or without fixation hooks, is discussed. Patients are advised about cosmetic optimization options for postoperative scar treatment.

Regarding the use of the robot, we explain to the patients that it is not an actual robot, but a precision instrument that is guided exclusively by the surgeon.

## Anesthesia and positioning

On the day of the operation, a final conversation is held with the patient in the day clinic, the hernia gap is marked on the skin with a felt-tip pen, and written consent is checked. The anterior abdominal wall is accessed from the left side of the patient, and the DaVinci Xi (Intuitive Surgical, Sunnyvale, CA, USA) is approached from the right side of the patient. The patient is positioned supine, aligned to the left border of the operating table (Trumpf Medical, Saalfeld, Germany), on an anti-slip mat (pink pad). The left arm is positioned slightly below the level of the table; the right arm is moved out for anesthesia (ipsilateral to the position of the DaVinci Xi), the face and ventilation tube are protected with a metal frame mounted on the operating table. The DaVinci Xi and the Trumpf table are coupled via Bluetooth and move synchronously. The procedure is performed under general anesthesia; relaxation must be optimal until the end of the procedure or until the robotic system is undocked; if needed, neuromuscular blockade is antagonized at the end of the procedure. Patients receive perioperative antibiotic prophylaxis with cefuroxime 1.5 g (alternatively clindamycin 600 mg).

In very small patients or with port positioning from caudal (e.g., access from the “bikini line”, see below), the flexion of the operating table must be taken into account during positioning. The physiological dorsal extension of the lumbar spine is 30–35°; on the Trumpf operating table, when the back plate is set to −20° (“re-flex”), it is comfortable and tolerable for the awake patient. Alternatively, extension in the hip joint is helpful, which is physiologically 15°; on the Trumpf operating table, with the leg plate set at −20°, it is comfortable and tolerable for the awake patient (because of the concomitant tilting of the pelvis and extension of the lumbar spine). Both positions provide sufficient freedom of movement from caudal for the robotic instruments.

## Overview of the relevant anatomy of the anterior abdominal wall

The *peritoneum* is perfused by numerous perforating vessels coming from the posterior rectus sheath. In the region of the anterior abdominal wall, the peritoneum embryologically covers the round ligament of the liver (the obliterated umbilical vein) with the falciform ligament in the supraumbilical region and median umbilical ligament (the obliterated urachus) as well as on both sides the lateral umbilical ligaments (corresponding to the pars occlusa of the respective umbilical arteries). These structures are embedded between the peritoneum and the abdominal wall in the preperitoneal adipose tissue and converge in the umbilicus, where they terminate blindly in the scarred attachment of the former umbilical cord at the umbilical base. Depending on the body structure, this preperitoneal adipose tissue extends to 1–4 cm lateral to the midline at the umbilicus; in the subxiphoidal area, it is up to 15 cm wide, and in the lower abdomen it opens into the space of Retzius [8]. The layer between the preperitoneal adipose tissue and the posterior surface of the linea alba is the plane for preperitoneal mesh implantation (plane “J”) [9]. When preparing the preperitoneal space around the umbilical hernial orifice, the ligaments ending therein must be cut so that the mesh can subsequently lie flat (without undulation) against the fascia endoabdominalis.

The vessels that supply the umbilicus in adults also transit through this area. The *blood supply to the umbilicus* is secured by multiple *collaterals* from both sides:

- By the subdermal plexus,
- The inferior epigastric arteries (which anastomose multiple times between right and left),
- By small vascular branches along the round ligament of the liver, and
- By vessels along the median umbilical ligament [10].

Studies from the field of plastic and reconstructive surgery have shown that the presence of an inferior epigastric artery on only one side is sufficient to provide blood flow to the umbilicus [10]. Branches of the inferior epigastric arteries reach the umbilicus from the rectus abdominis muscle and pass subcutaneously through the anterior rectus sheath as *perforating vessels*, where they anastomose with subcutaneous branches of the superficial epigastric vessels (arteries and veins, respectively). On average, there are 5.3 arterial perforator vessels around the umbilicus [11]. In the infraumbilical region, the venous outflow of the umbilicus is given by rich polygonal networks between both superficial epigastric veins [12].

The collagenous fibers of the *rectus sheath* join medially by decussation between the anterior and posterior sheaths into the linea alba. The posterior rectus sheath ends caudally on both sides respectively at the arcuate ligament; between the arcuate ligament and the pubic symphysis the rectal muscles are covered dorsally only by their own thin fascia, the fascia recti propria. During the preparation of the retrorectus space (level “F”) [9], the transition from the left to the right rectus sheath is made by detaching the respective lamina posterior approximately 3 mm lateral to the decussation plane; this creates a connection between both retrorectus spaces (plane “F”; [9]) and the preperitoneal space (plane “J”; [9]; see also Fig. 2c/3 below).

## Preperitoneal mesh implantation—access from left-lateral (online video 1/part 1)

**Fig. 1:**
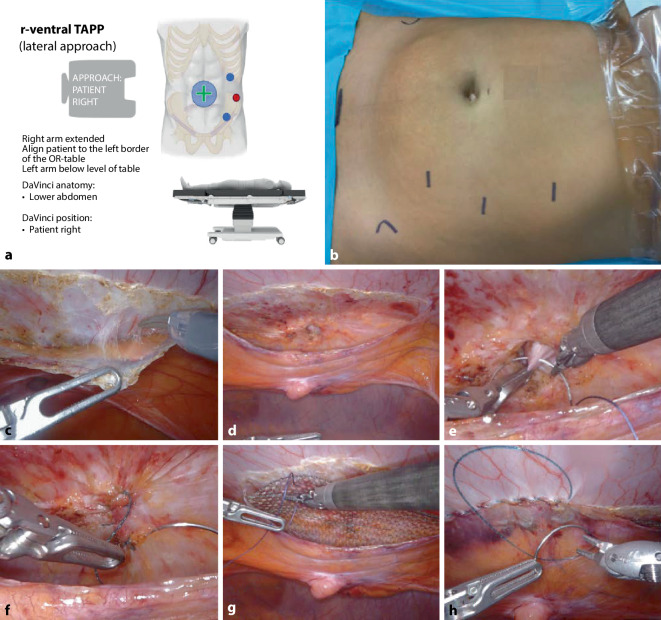
Robotic ventral transabdominal preperitoneal hernia repair (rv-TAPP) from left-lateral, in the example of an umbilical hernia. **a** Check-list for patient positioning, targeting and port placement. **b** Planning of port positions with marker pen. **c** Detachment of peritoneum from the fascia endoabdominalis. **d** End of parietalization on both sides of linea alba. **e** Reinsertion of umbilicus. **f** Transverse suture closure of hernia gap. **g** Mesh positioning and fixation. **h** Continuous suture closure of peritoneum

The ports are positioned on the left side of the body; the DaVinci Xi is on the right (setting on the DaVinci Xi patient cart: lower abdominal, patient on the right; Fig. 1).

Start with the WHO team time-out, followed by repetition of the surgical steps on the intraoperative checklist (supplementary online material 1). The pneumoperitoneum is created via Veres needle at left-subcostal site (12 mm Hg). After creating the pneumoperitoneum, the distance to the hernia is measured, the ports are positioned, and the anatomical nature of the umbilical plicae is inspected. If from supraumbilical and infraumbilical the plicae converge with abundant preperitoneal adipose tissue at the umbilicus (in umbilical hernia), or if in epigastric hernia it is surrounded by sufficient preperitoneal adipose tissue, the preperitoneal approach is usually well feasible. If the peritoneum is very thin and there is barely any preperitoneal adipose tissue, or if the hernia diameter exceeds 4 cm, there is an indication to extend the procedure to the retrorectus space for technical reasons (see below). In the following description of the technique, the umbilical hernia is taken as an example; epigastric hernias are prepared in an analogous manner.

With the Prograsp Forceps (the angled instrument measures 4 cm, the length of the non-isolated area is 4.5 cm) or with the ruler, the lateral preparation border is estimated and marked with monopolar coagulation points on the peritoneum to ensure sufficient mesh overlap later (supplementary online video 1, 01:08–01:20 min).

Access to the preperitoneal space is created parallel to the linea alba and at a lateral distance of approximately 5–6 cm from it over a length of approximately 12 cm (Fig. 1c). Dissection is performed between the peritoneum and endoabdominal fascia. When the linea alba is reached, it must be explored—especially cranial to the umbilicus—for concomitant additional findings in terms of asymptomatic epigastric hernias (additional online video 1 02:13–02:25 min). Before the umbilical hernia and the accompanying preperitoneal fat prolapse are recovered, the caudal dissection of the linea alba is recommended first for a better overview. As described above, the ligaments opening in the umbilicus are also transected. The accompanying preperitoneal fat body is retrieved in toto and the peritoneal hernial sac is carefully detached from the thin umbilical skin (caution: coagulation damage of the umbilical skin; Fig. 1/d).

After revision of hemostasis and measurement of the mesh size to be placed, reinsertion of the umbilicus is performed; the surgeon’s assistant (TOA) or assistant on the patient pushes the fundus of the umbilicus inward with the tip of the index finger, then it is grasped with a Vicryl suture (SH needle); the console surgeon removes the face from the immersion hatch of the DaVinci Xi, leaving the robotic arms immobile for the duration of the “absence” from the console (Fig. 1e); this allows the surgeon to check the configuration of the new umbilical fossa on the patient before finally fixing the umbilical base to the inferior border of the umbilical foramen.

If there is no rectus diastasis or the reefing of the entire linea alba has not been discussed with the patient, the hernial orifice is closed with a transverse suture using V‑Loc 3‑0 USP (Medtronic Germany) (additional online video 1 02:26–02:44 min; Fig. 1f). Subsequently, the flat large-pored mesh (Dynamesh Endolap Visible) is cut to the respective size and rolled up and inserted over the cranial port. This is followed by unrolling, positioning and fixation of the mesh with 4 loose corner sutures (absorbable suture material; additional online video 1 02:48–03:20 min; Fig. 1g). Finally, the peritoneum is closed with a continuous V‑Loc 3‑0 USP suture from caudal to cranial and the suture stump is extraperitonealized (Fig. 1h).

After checking the count of the surgical materials and revision of the surgical site, the procedure is terminated.

## Retrorectal mesh implantation—access from left-lateral (online video 2)

**Fig. 2:**
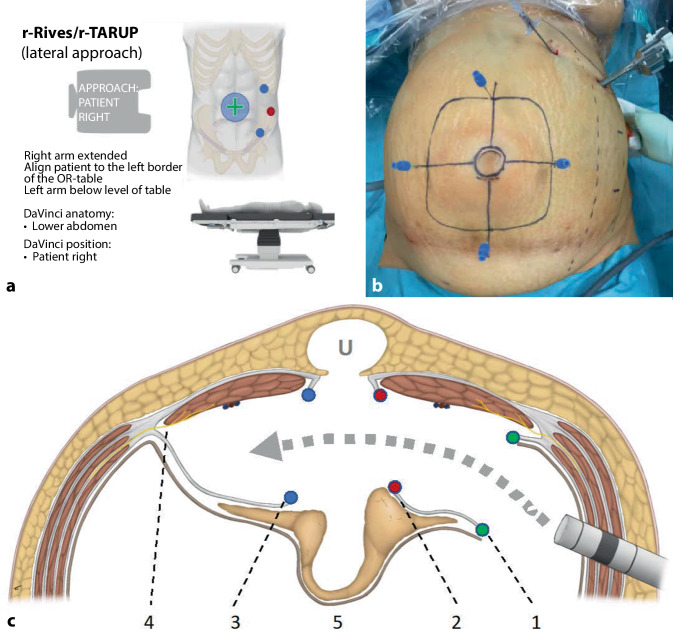
Planning of robotic transabdominal retromuscular umbilical prosthetic hernia repair (r-Rives or r‑TARUP) from left-lateral. **a** Check-list for patient positioning, targeting and port placement. **b** Pneumoperitoneum with Veres needle already done, umbilical hernia and planned mesh size marked with pencil and extrapolated to intra-abdominal with needles. **c** Anatomy of the r‑Rives/r-TARUP: the ports are positioned far lateral to the left of the rectus abdominis muscle, the *gray arrow* shows the path of dissection; *1* lateral entry into the left posterior rectus sheath (*green dots*); *2* in the area of decussation of both rectus sheaths, the posterior sheath is reopened (*red dots*), *U* umbilical hernia; *3* after exposing the linea alba and the hernial gap, the right posterior rectus sheath is entered medially (*blue dots*); *4* in the lateral region, the nerves must be preserved; *5* mobilized hernia sac with median peritoneal bridge between both posterior rectus sheaths

The ports are positioned on the left side of the abdomen; the DaVinci Xi is on the right (setting on the DaVinci Xi patient cart: lower abdomen, patient on the right; Fig. 2a).

Start with the WHO team time-out, followed by repetition of the surgical steps on the intraoperative checklist (supplementary online material 1). The pneumoperitoneum is created via the Veres needle left-subcostal (12 mm Hg). After creating the pneumoperitoneum, the distance to the planned port position from the hernia findings is measured and the lateral margin of the rectus abdominis muscle is estimated; alternatively, hernia gap and planned mesh size are drawn on the skin (Fig. 2b). Port positioning (2 × 8 mm, 1 × 12 mm): visual control of port depth at stationary point, exclusion of hemorrhage or intestinal lesion and adhesiolysis if necessary. To orient the internal extent of the preparation, the mesh size drawn on the skin is punctured with transparietal needles (Fig. 2b). Alternatively, lateral distance to the hernia can be measured with the Prograsp Forceps (the angled instrument measures 4 cm) or with the ruler to ensure adequate mesh overlap.

**Fig. 3:**
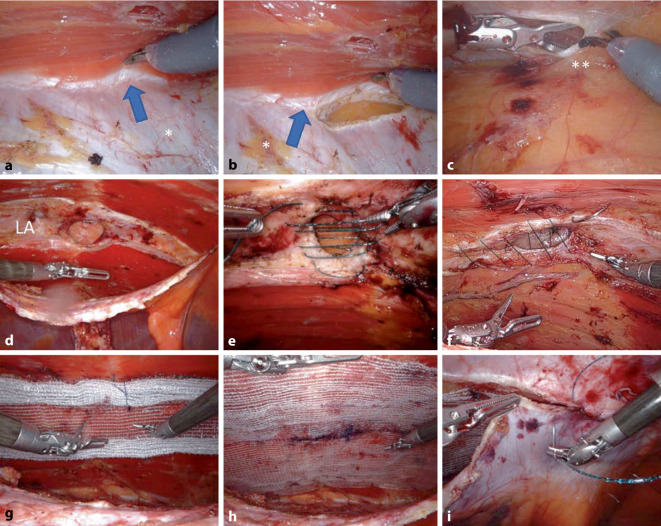
Robotic transabdominal retromuscular umbilical prosthetic hernia repair (r-Rives or r‑TARUP) from the left lateral side using recurrent umbilical hernia (incisional hernia) as an example.** a** Medial decussation (*blue arrow*) of both blades of the left rectus sheath (*asterisk*). **b** Opening of the posterior rectus sheath (*asterisk*) in the area of the linea alba (*blue arrow*). **c** Opening of the contralateral posterior rectus sheath close to the linea alba (**). **d** End of dissection, *LA* linea alba. **e** Example 1: transversal suture of the hernia gap. **f** Example 2: longitudinal suture of the hernia gap with tightening of the linea alba. **g** Positioning and rolling out of the mesh. **h** End of mesh positioning with wide overlap (mesh size in the image: 20 × 15 cm). **i** Running suture closure of the left posterior rectus sheath

Monopolar entry into the lateral border of the left posterior rectus sheath (Fig. 2c/1), parallel to the linea alba and 5–8 cm lateral to it (supplementary online video 2, 01:30–2:07 min); the rectus abdominis muscle is detached from the posterior rectus sheath with sparing monopolar dissection, preserving the epigastric vessels. The medial edge of the left-sided rectus sheath is reached (Fig. 2c/2 and Fig. 3a); the collagen fibers decussating between the posterior and anterior rectus sheaths form the linea alba, which is identified and preserved (supplementary online video 2, 02:23–02:43 min; Fig. 3a). Entry into the preperitoneal space through a second (medial) longitudinal opening of the posterior rectus sheath (Fig. 2c/2 and Fig. 3b). Caution: should the anterior rectus sheath be inadvertently opened, the dissection will mistakenly enter the subcutaneous tissue, the “Onlay” layer will be dissected, and the midline will become mechanically unstable.

The linea alba is now explored from cranial to caudal and the hernia gaps are expose; the hernia sac is parietalized and, together with the preperitoneal fat tissue, constitutes a bridge between both posterior rectus sheaths (Fig. 2c/5). The thin umbilical skin must be preserved. Upon reaching the right lateral border of the linea alba, the contralateral (right) rectus abdominis muscle is seen through the thin posterior rectus sheath of the opposite side (Figs. 2c/3 **and** 3c); the right posterior rectus sheath is also opened longitudinally parallel to the linea alba and detached from the rectus abdominis muscle (supplementary online video 2, 03:42–04:05 min). Lateral dissection should extend to at least 5–8 cm lateral to the hernial gaps; careful attention must be paid to the nerves running here that enter the rectus abdominis muscle from the lateral side, to avoid causing abdominal wall paralysis (Fig. 2c/4).

### Reinsertion of the umbilicus (in umbilical hernia).

If there is an umbilical hernia, the hypodermis of the umbilicus is now fixed to the caudal edge of the hernia gap with an absorbable suture (supplementary online video 2, 04:35–05:02 min); since this is an esthetic surgical step, the surgical team assures itself of the expected morphological result by inspecting the umbilicus on the patient before tying the suture.

### Transverse suture (Fig. 3e; supplementary online video 2, 05:03–05:58 min) or longitudinal suture (Fig. 3f) of the hernia gap.

In large umbilical hernias, the transverse suture is a good option because the shape of the abdomen is esthetically preserved; if the umbilical hernia is sutured longitudinally without concomitant gathering of the entire linea alba, the patient has a deformity of the abdomen postoperatively in the sense of a constriction, at the level of the umbilicus. Especially in slim patients, it is particularly recommended to close the isolated hernia gap transversely. In cases of concomitant rectus diastasis or multiple hernia gaps, two options should be considered: either the entire linea alba is sutured longitudinally, or the mesh is inserted as a bridging to avoid midline deformity; in obese patients, the linea alba can be sutured longitudinally in a stepwise fashion with V‑Loc 0 USP (30 cm) with good esthetic results (additional online video 2, 06:00–06:50 min). We use a Progrip mesh (Medtronic) tailored to the required size for the r‑TARUP, taking into account the recommendations of Tulloh and deBeaux [13]. The insertion, unrolling and positioning of the Progrip mesh are described in the video (Fig. 3g, h; supplementary online video 2, 06:51–08:28 min). We fixate all meshes (including the Progrip mesh) with loose corner sutures (absorbable suture material). Before suture closure of the posterior rectus sheath, the pneumoperitoneum is reduced to 8 mm Hg and hemostasis is controlled over a period of 2–3 min. The posterior rectus sheath is closed with a running suture with two V‑Loc sutures, one starting from the cranial and one starting from the caudal, both suture stumps are extraperitonealized (Fig. 3i). Check the peritoneum of the median line and close any preparation holes with Vicryl suture. After count check of the surgical materials and revision of the surgical site, the procedure is ended. The puncture site of the 12 mm port is closed with a transparietal suture.

## rv-TAPP and r-TARUP—access from caudal/suprapubic (online video 1/part 2 and video 3)

The ports are positioned suprapubically; the DaVinci Xi is on the right or left (setting on the DaVinci Xi patient cart: upper abdomen, patient on the right or left; special positioning with patient tilting up or hyperextension of the operating table; supplemental material 2, Fig. 4a).

**Fig. 4:**
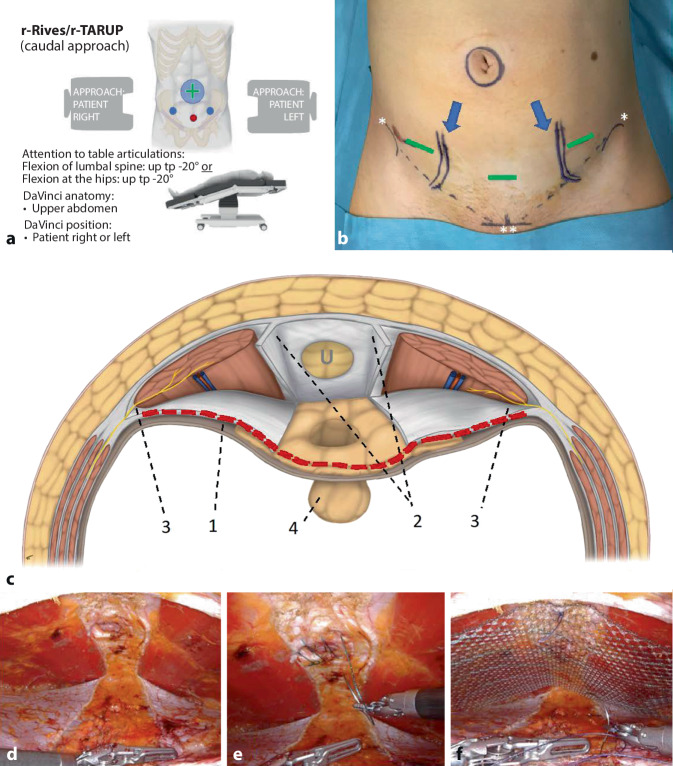
r‑Rives (robotic transabdominal retrorectus mesh positioning): caudal access to the retrorectus space, demonstrated by an incisional umbilical hernia. **a** Checklist for patient positioning, docking, and trocar placement. **b** Plot of the course of the inferior epigastric artery bilaterally (*blue arrows*) with ultrasound, for planning port positions (*green lines*). **c** Transverse section of the abdominal wall showing the caudal access to the retrorectus space, *U* umbilical hernia; *1* the *red dashed line* corresponds to the transverse incision of the abdominal wall to access the retrorectus space; *2* lead structure is the area of decussation of both rectus sheaths, the posterior rectus sheath blade is opened longitudinally parallel to the linea alba, the linea alba remains intact; *3* laterally, the nerve branches reach the rectus muscles; *4* retrieved fat prolapse with intact peritoneal bridge. **d** Operative site of the completed dissection. **e** Transverse suture closure of the hernial orifice. **f** Mesh positioning and fixation. (*Asterisk* anterior superior iliac spines; *double asterisk* pubic symphysis)

In selected patients with umbilical hernia (primary or recurrent), very small, slender or athletic body type, access from left-lateral may be difficult because of the required minimum distance between ports. In these cases, or for esthetic considerations to conceal incisions in the “bikini area,” the caudal approach has proven effective. Relative, technical contraindications, for this approach include a bulging abdomen and obesity, as the robotic arms cannot be placed in position to reach the internal abdominal wall from caudal under these conditions because the patient’s thighs and the anteroposterior diameter of the abdomen under pneumoperitoneum make the workspace inaccessible.

When planning port positions, attention must be paid to the course of the inferior epigastric vessels and the projection of the inguinal nerves (supplementary online video 3, 00:43–01:00 min; Fig. 4b). The former is done with Doppler ultrasound on the sterile skin of the abdomen or preoperatively with waterproof pen marking. At the level of the anterior superior iliac spine (ASIS), the epigastric vessels run posterior to the rectus abdominis muscle and in the region of its lateral third [14]; this vascular anatomy can be accurately visualized with Doppler ultrasound; the nerve course or passage through the abdominal wall is from lateral coming mediocaudal of the ASIS, so if the trocars are positioned 2 cm medial to the ASIS, they will not pass the rectus sheath but the lateral abdominal wall, albeit with low risk of nerve lesion. Start by positioning one of the lateral ports, then the median-suprapubic one to avoid lesion of the urinary bladder; since here median-suprapubic preperitoneal connective tissue is very elastic, trocar insertion may be technically difficult (counterpressure from inside with a laparoscopic clamp may be helpful).

The preperitoneal approach from caudal (rv-TAPP) is analogous to that described above—except for the entry (supplementary online video 1/part 2, 04:46–05:00 min; Fig. 4c–f). The retrorectus space (r-Rives) is accessed by transverse incision of both posterior rectus sheaths (supplementary online video 3, 01:19–02:00 min; Fig. 4c/1, red dashed line), with creation of the connection of both spaces by longitudinal incision of the medial insertion of both posterior rectus sheaths (supplementary online video 3, 02:02–02:38 min; Fig. 4c/2). The linea alba remains intact. The preparation can be extended from here to the xiphoid if necessary. Treatment of the hernia gap(s), umbilical skin, and principles of umbilical reinsertion or suture closure of the hernia gap(s) with or without median suture of the linea alba and mesh positioning and fixation are the same as described above for the lateral approach. A transverse continuous suture of both opened rectus sheaths or peritoneum is the final repair step.

## Patients and study design

This video article summarizes the experience of 118 consecutive surgeries, which were performed from June 2018 to December 2020. This is a comparative cohort study with 2 surgical procedures, and the choice of surgical procedure was based on intraoperative hernia findings. Data collection began with the first procedure of the implementation phase of the Visceral Surgery Robotics Program at Olten Cantonal Hospital and thus includes the period of the learning curve in the use of the surgical robot. The study was approved by the responsible ethics committee of Northwestern Switzerland (Ref. No. 2019-02046). Decisions on interventions at the level of the hernial orifices (type of suture of the hernial gap, refixation of the umbilicus, and exploration of the entire linea alba) were based on the respective findings. Patients were followed up 6 weeks postoperatively with clinical and sonographic evaluation, as needed. All data were recorded pseudonymously in an internal clinic database, which is password protected and accessible to the investigators. Patients generally remained inpatients for one night.

To compare the distribution of categorical variables, the χ^2^ test or the Fischer exact test were used, depending on the sample size; the t‑test was used for continuous variables. A *p* value less than 0.05 was considered significant.

## Results

A total of 88 patients underwent preperitoneal mesh implantation (rv-TAPP) and 30 underwent retrorectal mesh implantation (r-Rives or r‑TARUP). Patients in the r‑Rives group were significantly older (*p* = 0.001); there were no other demographic differences between the two groups by type of activity, comorbidities, or American Society of Anesthesiology (ASA) classification (Table 1).

**Table 1:** Demographic data

	Rv-TAPP (*n* = 88)		r‑Rives (*n* = 30)		*p*-value
**Age, mean (SD)**	52.3	(± 13.7)	62.1	(± 13.3)	**0.001**
**Female, *n* (%)**	25	(28.4)	15	(50.0)	**0.031**
**BMI kg/m**^**2**^**, mean (SD)**	30.7	(± 6.4)	29.2	(± 5.4)	0.250
**Smoker, *n* (%)**	37	(42.0)	14	(46.7)	0.659
**Ethnicity, *n* (%)**					
*Northern European*	70	(79.5)	24	(80.0)	0.957
*Mediterranean*	18	(20.5)	6	(20.0)	
**Type of professional activity, *n* (%)**					
*Desk-based*	20	(22.7)	3	(10.0)	0.062
*Physically demanding*	20	(22.7)	4	(13.3)	
*No work or retired*	16	(18.2)	12	(40.0)	
*Unknown*	32	(36.4)	11	(36.7)	
**Comorbidities, *n* (%)**					
*Arterial hypertension*	43	(48.9)	12	(40.0)	0.400
*Coronary heart disease*	10	(11.4)	3	(10.0)	0.836
*Diabetes mellitus*	13	(14.8)	7	(23.3)	0.280
*COPD*	8	(9.1)	5	(16.7)	0.252
*Thromboembolic event in anamnesis*	3	(3.4)	0	(0.0)	0.591
Deep vein thrombosis	3	(3.4)	0	(0.0)	0.305
Pulmonary embolism	1	(1.1)	0	(0.0)	0.557
*Immunosuppressive therapy*	2	(2.3)	0	(0.0)	0.405
*Oral anticoagulation*	16	(18.2)	6	(20.0)	0.825
DOAC	5	(5.7)	1	(3.3)	0.805
Coumarin	1	(1.1)	0	(0.0)	0.557
Platelet aggregation inhibitor	11	(12.5)	5	(16.7)	0.643
**ASA Score**					
*ASA I*	8	(9.1)	4	(13.3)	0.425
*ASA II*	64	(72.7)	18	(60.0)	
*ASA III*	16	(18.2)	8	(26.7)	

*SD* standard deviation, *ASA* American Society of Anesthesiology Score, *DOAC* dual oral anticoagulation, *BMI* body mass index, *COPD* chronic obstructive pulmonary disease, *r‑Rives* robotic transabdominal retrorectus mesh implantation [r-TARUP], *rv-TAPP* robotic ventral transabdominal preperitoneal mesh implantation

Both groups differed by type of hernia; primary ventral hernias were more commonly managed as rv-TAPP, and incisional hernias as r‑Rives (Table 2). In 4 patients in whom rv-TAPP was planned, r‑Rives was performed because the peritoneum was too thin; in one case, a 4 × 4 cm peritoneal tear was closed with a piece of Vicryl mesh in rv-TAPP; in none of the patients was it necessary to switch to r‑IPOM (robotic intraperitoneal onlay mesh). In 48 of the 118 patients, an additional finding was found intraoperatively at the linea alba that was asymptomatic (37.5%). The hernia gaps and the respective defect areas were significantly larger in the r‑Rives group (*p* < 0.001); analogously, the meshes were also significantly larger in the r‑Rives group (Table 2). The ratio of mesh area to hernia gap area was comparable in both groups (*p* = 0.142; Table 2). Mesh fixation was performed in 93% of patients in both groups, and subcutaneous drains were used in only 2 cases of large umbilical hernia (Table 2). The time to perform rv-TAPP was significantly shorter than to perform the r‑Rives, with an average of 82 min operating time (incision–suture time, including docking), compared with an average of 109 min (incision–suture time, including docking), respectively (Table 2). The time elapsed from Veres needle puncture (incision) to start at the console was 7–9 min.

**Table 2:** Characteristics of the hernias and procedures

	Rv-TAPP (*n* = 88)		r‑Rives (*n* = 30)		*p*-value
**Type of hernia, *n* (%)**					
*Primary umbilical*	53	(60.2)	10	(33.3)	**<** **0.001**
*Primary epigastric*	22	(25.0)	1	(3.3)	
*Primary lateral/Spieghel*	3	(3.4)	0	(0.0)	
*Incisional umbilical (EHS M3)*	4	(4.5)	2	(6.7)	
*Incisional (others) (EHS M2–M3–M4)*	6	(6.8)	17	(56.7)	
**Additional hernia gap at linea alba, *n* (%)**	38	(43.2)	10	(33.3)	0.797
**Size of hernial gap, mean (SD)**					
*Length, cm*	2.3	(±1.1)	4.9	(±1.1)	**<** **0.001**
*Width, cm*	2.2	(±1.0)	4.2	(±1.0)	**<** **0.001**
*Hernial orifice area, cm*^*2*^	8.8	(±9.4)	20.1	(±17.7)	**<** **0.001**
**Closure of hernial gap, *n* (%)**					
*None*	13	(14.8)	3	(10.0)	**<** **0.001**
*Longitudinal suture*	1	(1.1)	8	(26.7)	
*Transversal suture*	74	(84.1)	19	(63.3)	
**Size of the mesh, mean (SD)**					
*Length, cm*	11.6	(±3.5)	16.1	(±4.0)	**<** **0.001**
*Width, cm*	9.0	(±2.1)	12.7	(±2.9)	**<** **0.001**
*Area of the mesh, cm*^*2*^	107.8	(±56.0)	205.5	(±77.6)	**<** **0.001**
**Mesh area:hernial gap area ratio**	30.1	(±50.1)	16.5	(±12.8)	0.142
**Type of mesh, *n* (%)**					
*Dynamesh Endolap Visible*	73	(83.0)	2	(6.7)	**<** **0.001**
*Progrip*	15	(17.0)	27	(90.0)	
*Symbotex*	0	(0.0)	1	(3.3)	
**Mesh fixation, *n* (%)**					
*None*	1	(1.1)	0	(0.0)	0.828
*Vicryl suture*	82	(93.2)	28	(93.3)	
*V‑Loc suture*	5	(5.7)	2	(6.7)	
**Subcutaneous drain, *n* (%)**	2	(2.3)	0	(0.0)	0.405
**Total operative time (in min), mean (SD)**	82.9	(±21.0)	109.1	(±32.4)	**<** **0.001**

*SD* Standard deviation, *EHS* Classification of the European Hernia Society (Muysoms et al.), *r‑Rives* robotic transabdominal retrorectus mesh implantation [r-TARUP], *rv-TAPP* robotic ventral transabdominal preperitoneal mesh implantation

Hospital stay was shorter in the rv-TAPP group than in the r‑Rives group (1.5 vs. 2.7 days, respectively; *p* < 0.001; Table 3). There was no difference in the incidence of seroma, hematoma, or skin necrosis; overall, there was no difference in the incidence of adverse events between the two groups; the only difference was a significant clustering of type II seromas in the r‑Rives group (*p* < 0.001), but the total number of seromas was comparable (Table 3).

**Table 3:** Postoperative course

	Rv-TAPP (*n* = 88)		r‑Rives (*n* = 30)		*p*-value
**Outpatient procedure, *n* (%)**	15	(17.0)	3	(10.0)	0.354
**Length of hospital stay, days, mean (SD)**	1.5	(±0.6)	2.7	(±1.7)	**<** **0.001**
**VAS score on postoperative day 1, mean (SD)**^**a**^	2.3	(±2.0)	2.6	(±1.5)	0.529
**Adverse events**					
*Surgical site occurrence (SSO), n (%)*	16	(18.2)	9	(30.0)	0.171
Seroma (nach Morales–Conde), *n* (%)	14	(15.9)	7	(23.3)	0.358
– Grade I	1	(1.1)	–	–	**<** **0.001**
– Grade II	11	(12.5)	5	(16.7)	
– Grade III	2	(2.3)	0	(0.0)	
– Grade IV	–	–	2	(6.7)	
Hematoma, *n* (%)	3	(3.4)	3	(10.0)	0.155
Skin necrosis, *n* (%)	–	–	1	(3.3)	0.085
*Unscheduled presentation due to pain*	5	(5.7)	1	(3.3)	0.613
*Delayed onset of intestinal transit, n (%)*	1	(1.1)	1	(3.3)	0.557
*Pulmonary embolism, n (%)*	2	(2.3)	–	–	0.405
*Clavien–Dindo, n (patients)*					
Grade I	23	(20)	9	(8)	0.661
Grade II	2	(2)	–	–	0.405
Grade III	–	–	1	(1)	0.085
Grade IV	–	–	1	(1)	0.085
CCI, mean (SD)	2.7	(±5.6)	4.4	(±8.1)	0.191
**Follow-up after 6 weeks, *n* (%)**					
*Done*	74	(84.1)	28	(93.3)	0.201
*Recurrence*	–	–	–	–	1.000
*Abdominal wall pain*	5	(6.7)	–	–	0.161
*Seroma*	10	(13.3)	7	(25.0)	0.155
*Hematoma*	1	(1.3)	1	(3.7)	0.446

*SD* Standard deviation, *VAS* Visual analog scale (from 1, no pain to 10, worst pain), *CCI* Charlson Comorbidity Score, *SSO* surgical site occurrence, *r‑Rives* robotic transabdominal retrorectus mesh implantation [r-TARUP], *rv-TAPP* robotic ventral transabdominal preperitoneal mesh implantation

^a^For patients with hospital stay

In the closer analysis of wound complications (surgical site occurrence [SSO]), with comparison of SSO+ or SSO−, both procedures were comparable. Strikingly, in this subgroup analysis, age, BMI, and nicotine use had no negative effect on outcome. Also, the ratio of the mesh area to the hernia gap area did not correlate with SSOs (Table 4).

**Table 4:** Characteristics of patients with surgical site occurrence

	SSO −		SSO +		*p*-value
	(*n* = 93)		(*n* = 25)		
**Age, mean (SD)**	54.1	(±14.2)	57.6	(±14.1)	0.265
**Female, *n* (%)**	29	(31.2)	11	(44.0)	0.229
**BMI kg/m**^**2**^**, mean (SA)**	29.9	(±6.2)	31.8	(±5.8)	0.179
**Smoker, *n* (%)**	38	(40.9)	13	(52.0)	0.318
**Comorbidities, *n* (%)**					
*Arterial hypertension*	42	(45.2)	13	(52.0)	0.542
*Coronary heart disease*	8	(8.6)	5	(20.0)	0.106
*Diabetes mellitus*	14	(15.1)	6	(24.0)	0.289
*COPD*	11	(11.8)	2	(8.0)	0.587
*Oral anticoagulants*	18	(19.4)	4	(16.0)	0.702
*ASA Score*					
– ASA I	11	(11.8)	1	(4.0)	0.347
– ASA II	65	(69.9)	17	(68.0)	
– ASA III	17	(18.3)	7	(28.0)	
**Type of hernia, *n* (%)**					
*Primary umbilical*	50	(53.8)	13	(52.0)	0.764
*Primary epigastric*	17	(18.3)	6	(24.0)	
*Incisional*	23	(24.7)	6	(24.0)	
*Spieghel*	3	(3.2)	–	–	
**Additional hernia gap at linea alba, *n* (%)**	34	(36.6)	13	(52.0)	0.567
**Hernial gap area cm**^**2**^**, mean (SA)**	10.6	(±11.5)	15.7	(±17.2)	0.085
**Procedure, *n* (%)**					
*Rv-TAPP*	72	(77.4)	16	(64.0)	0.171
*r‑Rives*	21	(22.6)	9	(36.0)	
**Closure of hernial gap, *n* (%)**	19	(20.4)	8	(32.0)	0.221
**Area of the mesh cm**^**2**^**, mean (SA)**	132.9	(±77.8)	131.9	(±65.8)	0.953
**Mesh area:hernial gap area ratio**	29.7	(±48.7)	14.9	(±10.3)	0.143
**Type of mesh, *n* (%)**					
*Dynamesh*	61	(65.6)	14	(56.0)	0.553
*Progrip*	31	(33.3)	11	(44.0)	
*Symbotex*	1	(1.1)	–	–	
**Total operative time**^**a**^** (in min), mean (SD)**	88.9	(±26.8)	91.9	(±27.3)	0.625

*ASA* American Society of Anesthesiology Score, *COPD* chronic obstructive pulmonary disease, *BMI* body mass index, *CCI* Charlson Comorbidity Score, *SSO* surgical site occurrence, *r‑Rives* robotic transabdominal retrorectus mesh implantation [r-TARUP], *rv-TAPP* robotic ventral transabdominal preperitoneal mesh implantation

^a^Total operative time includes the time elapsed from beginning of pneumoperitoneum, docking, undocking and suture of the skin

## Discussion

The ideal repair of median abdominal hernias has not yet been established. However, data from the past few decades have provided a good overview of the advantages and limitations of individual procedures:

- BMI, age, and nicotine use are the most significant risk factors for complications,
- Hernia orifice size and mesh overlap correlate with recurrence,
- Open procedures have more complications and fewer recurrences, minimally invasive procedures fewer complications and more recurrences, and
- Hematomas and seromas occur with similar frequency in the different procedures [2, 4, 13, 15–17].

A recent Delphi study recapitulated the layers of the abdominal wall available for mesh repair and clarified the multiple options for mesh positions [9]. While in the past two decades laparoscopic repairs were mostly performed as an IPOM technique, with or without hernia gap closure (using meshes in contact with the abdominal organs), the current trend is to minimally invasively transfer meshes from the abdominal cavity to one of the various available layers. A pioneer of this idea was Marc Miserez of the University of Leuven in Belgium, who described an endoscopic total extraperitoneal procedure (by today’s definition a retrorectus repair) for ventral hernia in 15 patients as early as 2002 [18]. Later, the transabdominal approach to the retrorectus space was also described [19]. The benchmark against which any repair of umbilical hernias must be measured is probably the E/MILOS technique, in which a cohort study of 520 umbilical hernias described near-ideal results of 1.2% complications, 0.0% infections, and 0.0% recurrences at one year; chronic pain requiring treatment occurred in 0.6% of patients [3]. However, whether every umbilical hernia requires such a large mesh as is common with E/MILOS remains to be debated, not least because younger patients in particular can expect to undergo abdominal surgery again later in life, and larger-than-necessary meshes can become problematic for access to the abdomen. The data on E/MILOS still need to be externally validated by other centers and verified in randomized controlled trials in the future.

The current consensus seems to be that modern procedures must combine the advantages of open repair (morphologic and functional repair, extraperitoneal mesh, low recurrence rate) with those of minimally invasive procedures (fewer complications) [20]. The conventional laparoscopic linea alba stapler repair (LIRA) procedure is a step in this direction; the hernia gap is closed at the fascial level, but the mesh is still implanted in the IPOM position [21].

With robotics, the goal of extraperitoneal mesh with morphologic reconstruction and minimally invasive procedure has come closer in a new quality. Nadia Henriksen from Copenhagen, Denmark, has shown in a meta-analysis that robotics can be beneficial in ventral hernias [22]. The current cohort study investigates two robotic procedures: transabdominal preperitoneal mesh implantation (rv-TAPP) and transabdominal retrorectus mesh implantation (r-Rives or r‑TARUP). Kudsi et al. have shown that rv-TAPP has a learning curve of approximately 46 patients by which time peritoneal detachment is technically optimized, with reduction of peritoneal tears from 63% to 11%; the average operating time was 54 min in 105 patients [23]. In our study, the operating time was longer, 82 min, although the size of the hernia lacunae was comparable (Table 2). This is likely due to the fact that, working with two consoles, every procedure in our study was a training procedure for residents; however, our data show that even under training conditions, a stable surgical time of well under 90 min can be maintained. In contrast to the study by Kudsi et al., the ratio of mesh area to hernia gap area in our study is higher [2, 19], with a mean of 30.1, which may also have contributed to the longer operative time (Table 2; [23]). The preperitoneal approach is the least traumatic to the abdominal wall; disadvantageously, the wide-area detachment of the sometimes thin but very well perfused peritoneum may complicate the procedure for meshes over 10 cm in width, so for hernias over 4 cm in diameter, the retrorectus space should be preferred.

We perform the retrorectus repair (r-Rives or r‑TARUP) in analogy to the technique described by Muysoms [24]. Similar to Muysoms’ cohort, most hernias in our cohort are of umbilical topography; in Muysoms, 7 of 42 were incisional, whereas in our cohort, 20 of 30 were incisional (Table 2; [24]). In our cohort, in patients treated as r‑Rives with hernia areas around 20 cm^2^, an average mesh area to hernia area ratio of 16.5 was achieved, which fulfills the theoretical requirements of Tulloh and deBeaux (Table 2; [13]). Postoperative complications were low (Table 3).

The number of hernia gaps of the linea alba that are asymptomatic but seen intraoperatively as additional findings has not been described in the literature—not in classic textbooks or in a recent PubMed search. The 37.5% described here is published for the first time and demonstrates the usefulness of exploring the linea alba in the vicinity of symptomatic hernia findings, which is not possible with open periumbilical approaches.

Robotic surgical access from caudal (described above for both the preperitoneal and retrorectus spaces) is useful for hernias of the umbilicus and supraumbilical linea alba for two reasons: (a) additional findings in the sense of primarily ventral hernias rarely occur infraumbilically (hypogastric hernias have not been described), so that sufficient distal overlap of the mesh beyond the main finding is possible from the caudal approach [25]; (b) even if longitudinal tightening of the linea alba (in case of concomitant rectus diastasis) is planned, the suture in the infraumbilical region is rarely necessary up to the symphysis, because infraumbilical (physiological) diastasis is practically absent. This is explained by Ranney, who postulates that the superior region of the linea alba responds dynamically to pressure changes (respiration and feeding), whereas the infraumbilical region of the linea alba serves exclusively to stabilize the weight of the intra-abdominal organs and is thus much less distensible than the superior region of the linea alba [25]. We have performed the caudal approach in 6 patients to date.

The classic risk factors for complications (age, BMI, nicotine, comorbidities) did not correlate with more complications in this cohort; this may mean that robotics may be particularly important in high-risk patients; it would be worthwhile to collect more data here. For hernia gap area alone, a trend for more wound complications was found for larger hernias, however with no statistical relevance (Table 4). In cases of morbid obesity, it may be appropriate to perform bariatric surgery prior to hernia repair [17, 26].

For hernias larger than 8 cm in diameter, the procedures described here are not indicated because adequate mesh underfilling is not possible. Larger hernias are managed robotically as transversus abdominis release (r-TAR), a procedure originally described by Alfredo Carbonell for open surgery as posterior component separation and reviewed for robotic use in the third article of this series in *Der Chirurg* [27].

Finally, a word on the question of cost: the r‑Rives or r‑TARUP costs 1330 CHF (of which 950 CHF for the DaVinci material under the Extended Use Program and 380 CHF for the mesh); in comparison, laparoscopic IPOM with comparative hernia size incurs costs of 2380 CHF (of which 1380 CHF is for the mesh, 720 CHF for 2 staplers and 280 CHF for the disposable trocars); the laparoscopic IPOM costs 2330 CHF (of which 1380 CHF is for the DaVinci material under the Extended Use Program and 380 CHF for the mesh); in comparison, laparoscopic IPOM with comparative hernia size incurs costs of 2330 CHF (of which 1380 CHF is for the mesh, 720 CHF for 2 staplers and 280 CHF for the disposable trocars); the laparoscopic IPOM costs 950 CHF more than the robotic procedure in our hospital (the exchange rate to the USD is transferable at approx. 1:1). If the 420 CHF reallocation of the robot maintenance flat rate (for 300 procedures/year) per patient is taken into account, the robotic procedure achieves savings of 630 CHF per case within the diagnosis-related groups (DRG) remuneration, compared to the laparoscopic IPOM. The cost advantages of less postoperative pain (no staples and no transparietal sutures) and shorter hospital stay are not included in this analysis.

In summary, robotic technology allows safe and new minimally invasive approaches to the different layers of the abdominal wall and in the vast majority of cases allows the extraperitonealization of meshes, with a low complication rate. This development is the natural progression of the knowledge gained from 30 years of laparoscopy and the beginning of a new era.

## Keypoints for practice

In approximately one-third of patients with a primarily ventral hernia, a second, concomitant, asymptomatic hernia of the linea alba is found in addition to the main finding.

Robotic repair of ventral and incisional hernias:

- Has all the advantages of minimally invasive procedures (low complication rate).
- Integrates advantages of open procedures (morphologic reconstruction).
- Allows consistent extraperitonealization of meshes.
- Is a very flexible instrument for tailored approach: umbilical and epigastric hernias (< 4 cm) are treated as rv-TAPP (robotic ventral transabdominal preperitoneal hernia repair); incisional hernias, large hernia gaps (4–7 cm) and in case of planned tightening of the linea alba, the r‑Rives or r‑TARUP (robotic transabdominal retromuscular umbilical prosthetic hernia repair) is performed.
- Allows individual port positioning, depending on the type and location of the hernia.
- Is a suitable procedure for training of residents.
- Is less expensive than conventional laparoscopic IPOM (intraperitoneal onlay mesh).

## Supplementary Information

Video 1 rv-TAPP (parts 1 and 2)

Video 2 TARUP I (left lateral)

Video 3 TARUP II (caudal)

Supplemental material 1 Intraoperative checklist

## Declarations

The data collection of this study is based on a positive ethics vote of the Ethics Committee Northwestern Switzerland (No. 2019-02046).
