# Erratum to: Robotic hernia repair II. English version: Robotic primary ventral and incisional hernia repair (rv-TAPP and r-Rives or r-TARUP). Video report and results of a series of 118 patients

**DOI:** 10.1007/s00104-021-01563-x

**Published:** 2021-12-20

**Authors:** Johannes Baur, Michaela Ramser, Nicola Keller, Filip Muysoms, Jörg Dörfer, Armin Wiegering, Lukas Eisner, Ulrich A. Dietz

**Affiliations:** 1 Department of Visceral, Vascular and Thoracic Surgery, Cantonal Hospital Olten (soH), Baslerstraße 150, 4600 Olten, Switzerland; 2 Department of General, Visceral and Vascular Surgery, Cantonal Hospital Baden, Im Engel 1, 5404 Baden, Switzerland; 3 Department of Surgery, AZ Maria Middelares, Buitenring Sint-Denijs 30, 9000 Ghent, Belgium; 4 Department of General, Visceral, Transplant, Vascular and Pediatric Surgery, University Hospital Wuerzburg, Oberduerrbacher Str. 6, 97080 Wuerzburg, Germany

**Erratum to:**

**Chirurg 2021**

10.1007/s00104-021-01479-6

The article “Robotic hernia repair. rv-TAPP and r‑Rives or r‑TARUP” is part II and a translation of the originally in *Der Chirurg *9/21 published German language article. The title and subtitle have been amended accordingly. The original article has been corrected.

