# Supplementary material for: Robotic hernia repair II. English version: Robotic primary ventral and incisional hernia repair (rv‑TAPP and r‑Rives or r‑TARUP). Video report and results of a series of 118 patients
Source: Chirurg. 2021 Aug 10;92(Suppl 1):15–26. doi: 10.1007/s00104-021-01479-6 (PMC8695563; doi:10.1007/s00104-021-01479-6)
Supplement: Supplementary file 4 — Supplemental material 1 Intraoperative checklist [file 104_2021_1479_MOESM4_ESM.pdf]

Patient ID

Date: _____/_____/_____

Surgeon: _____________

rv-TAPP/r-Rives
(lateral approach)

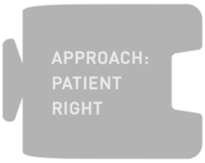

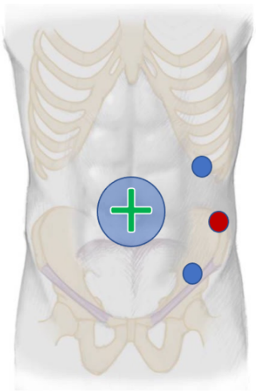

Right arm extended
Align patient to the left border of the OR-table
Left arm below level of table

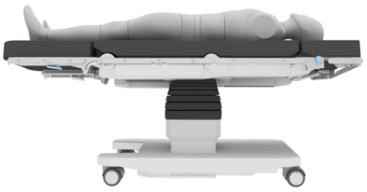

DaVinci anatomy:
- Lower abdomen

DaVinci position:
- Patient right

Perioperative Procedures/daVinci Checklist (Note: Does not replace the WHO Team Time-Out).

I have reviewed the patient informed conset sheet and checked it for content and comments: □ Yes □ No
Perioperative antibiotics: □ Yes □ Cefuroxim 1,5g to 3g (Alternative: □ Clindamycin 600mg)
Hybrid-Procedure: □ Yes □ No If yes, inform nurse to prepare additional instruments.

<u>Before docking</u>: Distance of trocar line to symphysis is approximately 20cm: □ Yes □ No
It is a large scrotal hernia, scrotum should be disinfected: □ Yes □ No
Previous prostate radiation or prostate pre-surgery: □ Yes □ No
It is a recurrence: □ Yes □ No
If yes, senior surgeon was informed prior to start of surgery: □ Yes □ No
Ropivacaine infiltration of port incisions: □ Yes □ No
Laparoscopic visual inspection of port placement □ Yes □ No

<u>Console</u>: Complete adhaesiolysis of the abdominal wall: □ Yes □ No

r-Rives (Synonym: r-TARUP):
Entry into ipsilateral rectus sheath, far lateral: □ Yes □ No
Caveat: Crossover at medial border of the rectal sheath: second incision of the posterior rectus sheath, the linea alba should not be damaged.
Caveat: Instability of the linea alba if accidental entry through the anterior rectus sheath.
Caveat: Do not damage the thin umbilical skin.
Complete mobilisation of fatty tissue from hernia gaps: □ Yes □ No
Exclusion of further hernias up to the xiphoid: □ Yes □ No
Präparation in the spaces of Retzius- and Bogros needed: □ Yes □ No
Umbilical pit was reinserated with absorbable suture: □ Yes □ No
Transverse V-Loc-suture of the hernial gap: □ Yes □ No
Mesh-Measuring in cm; Fixation with loose absorbable stitches: □ Yes □ No
Running suture of ipsilateral posterior rectus sheath (V-Loc 3-0): □ Yes □ No

rv-TAPP:
As r-Rives, incise peritoneum to entry the preperitoneal plane: □ Yes □ No
Accidental tears of the peritoneum were sutured: □ Yes □ No
Alternative: Vicryl mesh interposition if large peritoneal tears: □ Yes □ No

Finishing:

Laparoscopic visual inspection of port removal:	□ Yes	□ No
If 12mm port was used: transparietal suture closure performed:	□ Yes	□ No
OR data entered into registry:	□ Yes	□ No
Normal postoperative prescription:	□ Yes	□ No

Pre-stationary preparation:

CT abdomen with transverse and
and sagittal sections (morphology and adhesions)
Würzburg incisional hernia classification
If hernia gap is more than 8cm wide, consider r-TAR or r-IPOM.

Sutures and materials:

V-Loc 180 (green) 3-0 USP V-20 (15cm)
Vicryl 3-0 SH (20cm)
Vicryl 3-0 without needle For mesh rolling up
Ruler
DynaMesh Endolap Visible 15x10cm mesh
(If necessary: Vicryl mesh 28x18cm)
(If necessary: Progrip mesh 30x15cm)
Marking pen

Positioning of the patient on OR-table:

Supine position
No pink pad needed
Right arm is extended
Patient lies flush left on operating table
Left arm slightly below body level

Planned OP time: 60-120min

Instruments:

30° optics
Prograsp Forceps
Mega SutureCut Needle Driver
Hot Shears MCS
3x 8mm daVinci Trocars
(event. 1x 12mm daVinci Trocar)

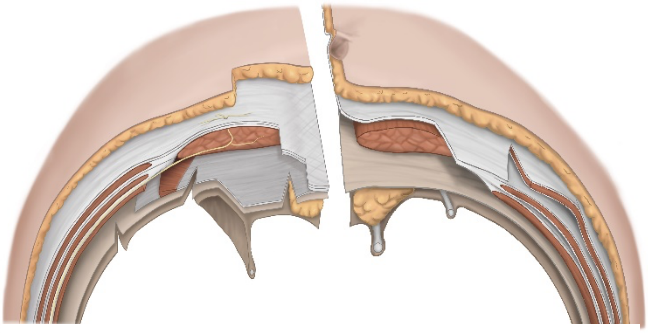

Supraumbilical planes **Infraumbilical planes**

From: Dietz, Fortely, Beldi, Wiegering (Eds.) Offene Hernienchirurgie (2012) Springer Verlag

Skin closure:

Ropivacaine 0,5% 10ml
Caprosyn 4-0 USP with P-24 needle
Cyanoacrylate-Glue

Postoperative prescription:

Mobilisation on the same evening
No limitations for physical activities after day 3

CIRS-report: □ Yes □ No

Swiss DRG G09D, Target dwell time: **0** | 3 | 9 days

Planned discharge: _____/_____/_____

Ocurrence	M = morphology	S = size (cm)	RF = risk factors (+ → +++)
p *(primary)*<br>r *(recurrent)*	m *(median)*<br>u *(umbilical)*<br>sp *(suprapubic)*<br>sc *(subcostal)*<br>t *(transversal)*<br>l *(lumbal)*<br>pm *(paramedian)*<br>nc *(other)*	a x b	Obesity (BMI >25)<br>Male gender<br>Nicotine abuse<br>Wound contamination<br>Age > 45 years<br>Disease<br>2nd laparot. < 1 month<br>Emergency procedure<br>Postop. Complications<br>…

v/i M… S…x… RF…

Debriefing: What can be done better in the next surgery?
